# Modeling and Simulation of the Impact of Feed Gas Perturbation on CO_2_ Removal in a Polymeric Hollow Fiber Membrane

**DOI:** 10.3390/polym14183783

**Published:** 2022-09-09

**Authors:** Nayef Ghasem

**Affiliations:** Department of Chemical & Petroleum Engineering, United Arab Emirates University, Al-Ain City P.O. Box 15551, United Arab Emirates; nayef@uaeu.ac.ae

**Keywords:** CFD simulation, membrane contactor, CO_2_ removal, oscillating flow, overall mass transfer coefficient, potassium glycinate

## Abstract

A membrane contactor is a device that attains the transfer of gas/liquid or liquid/liquid mass without dispersion of one phase within another. Membrane contactor modules generally provide 30 times more surface area than can be achieved in traditional gas absorption towers and 500 times what can be obtained in liquid/liquid extraction columns. By contrast, membrane contactor design has limitations, as the presence of the membrane adds additional resistance to mass transfer compared with conventional solvent absorption systems. Increasing mass transfer in the gas and solvent phase boundary layers is necessary to reduce additional resistance. This study aims to increase the mass transfer in the gas phase layer without interfering with membrane structure by oscillating the velocity of the feed gas. Therefore, an unsteady state mathematical model was improved to consider feed gas oscillation. The model equation was solved using Comsol Multiphysics version 6.0. The simulation results reveal that the maximum CO_2_ removal rate was about 30% without oscillation, and at an oscillation frequency of 0.05 Hz, the CO_2_ percent removal was almost doubled.

## 1. Introduction

Power plants established on fossil fuels are the main source of CO_2_ emissions and hence, a primary environmental and climate change concern [1]. Reducing CO_2_ emissions from power plants through carbon capture is a reasonable solution. Various CO_2_ capture techniques have been implemented, including cryogenic separation, solvent absorption, and, lately, membrane solvent-gas contactors [2,3,4]. Membrane solvent-gas technology has the advantages of gas separation and compact sizes with resealable CO_2_ absorption [5,6]. By contrast, membrane separation technologies introduce mass transfer resistances in the liquid phase, gas phase, and membrane layer [7]. Membrane contactors are also used for purposes other than absorption. Membrane contactors have been used to strip dissolved gases other than CO_2,_ such as methane from wastewater treatment anaerobic effluents, natural gas, and flue gas [8,9]. The treatment method has been used in converting chemical oxygen to biogas. As methane is the primary source of biogas, the membrane separation process is utilized to liberate dissolved methane from anaerobic effluents [10]. Potassium glycinate solvent is an effective solvent as an aqueous solution used to absorb CO_2_ inside a solvent-gas hollow fiber membrane contactor [11,12]. Feed gas oscillating flow was induced experimentally, and the mass transfer correlations were improved in terms of Sherwood number. The study revealed that inducing mixing with the gas phase enhanced the absorption rate in the membrane contactor [13]. Thin polymeric hollow fiber films are another type of practical hollow fiber membrane for CO_2_ removal [14,15].

Computational fluid dynamics (CFD) and the design of experiments were performed on a membrane distillation. Simulation results revealed that the membrane module’s length strongly impacts the flux [16]. Mathematical modeling was performed for membrane contactors for CO_2_ absorption in different solvents. Various solvents were used in the CO_2_ absorption process, such as 4-diethylamino-e-butanol (DEAB), diethanolamine (DEA), and methyl diethanolamine (MDEA) in hollow fiber modules. DEAB absorption rate was competitive with MEA aqueous solutions [17]. The gas velocity in membrane gas absorption plays a significant role in improving separation performance, and the addition of baffles improves SO_2_ absorption performance at the critical velocity [18].

The ammonia-based CO_2_ capturing process in a hollow fiber membrane contactor shows better mass transfer performance in the dense membrane phase than conventional gas/liquid absorption processes [19]. The membrane outer diameter has a substantial impact on membrane separation performance. Membrane modules with fibers finer than 1.2 mm in diameter increased mass transfer resistance and decreased the membrane contactor specific area per unit volume. A membrane with a 0.9 mm outer diameter or less was more suitable [20]. A significant parametric study for different physical solvents revealed the importance of physical solvents in absorption and regeneration processes compared with the conventional packed column technology [21]. Developing gas-liquid membrane conductors reduces capital cost and energy consumption of conventional CO_2_ absorbers and separation columns [22,23,24]. The membrane material, type of solvent, configuration of solvent and gas placed in the lumen or shell side, and the operating temperature plays significant roles in conquering the technical challenges of membrane wetting and may result in a decrease in the mass transfer coefficient [25,26]. Nanoparticles such as SiO_2_ and Al_2_O_3_, along with MEA aqueous solution, help reduce the energy consumption of CO_2_ stripping. The highest mass transfer resistance is in the liquid phase, and increased liquid velocity increases the desorption flux [27].

Based on the above findings, there are difficulties in increasing the mass transfer coefficient based on the membrane structure, composition, and configuration. Accordingly, the current work focused on modeling and simulating the gaseous solvent membrane contactor based on the oscillating feed gas flow rate to increase CO_2_ removal without affecting membrane structure and composition.

## 2. Mathematical Model

**Figure 1:**
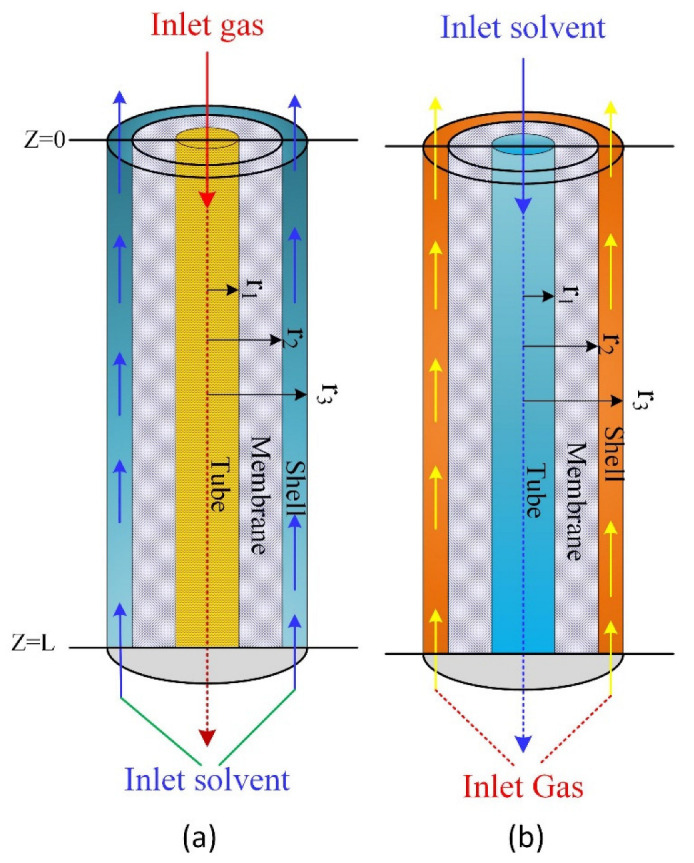
Schematic diagram and the membrane contactor model domain: (**a**) gas fed in the lumen side, (**b**) gas fed in the shell side.

We built a two-dimensional unsteady state mathematical model to describe carbon dioxide absorption from methane in potassium glycinate (PG) aqueous solution using a hollow fiber membrane absorption process under the influence of an oscillating gas flow. The model was solved in two scenarios; the first method is to validate the model predictions in which the gas is fed into the tube lumen side (Figure 1a). In the second scenario, the gas is fed to the shell side (Figure 1b). The model equations listed below describe the most common scenario taking place in a membrane contactor. The potassium glycinate solvent is fed to the lumen at z = L, and the gas stream enters the shell side currently at z = 0 (Figure 1b). Specific assumptions were considered, such as isothermal operation, laminar flow, and Henry’s law, which is applicable at the liquid-gas interface and non-wetting mode (gas-filled membrane pores).

### 2.1. Hollow Fiber Lumen (Solvent Flow)

Considering the case where gas is fed into the shell side and liquid solvent to the lumen side, the unsteady steady-state gas transport in the lumen solvent side i is ${\mathrm{CO}}_{2},\;{\mathrm{CH}}_{4}$.

(1) $$\frac{\partial C_{i,t}}{\partial t}=-D_{i,t}\left[\frac{\partial }{\partial r}\left(\frac{1}{r}\frac{\partial C_{i,t}}{\partial r}\right)+\frac{\partial^{2}C_{i,t}}{\partial z^{2}}\right]+r_{i,t}+v_{z,t}\frac{\partial C_{i,t}}{\partial z}$$

where $c_{i,t}$ is the molar concentration of CO_2_ in the liquid solvent flowing in the membrane tube side and $D_{i}$ is the diffusion coefficient of CO_2_ in the tube side.

The aqueous solution velocity ($v_{z,t}$):

(2) $$v_{z,t}=\frac{2Q_{t}}{n\pi r_{1}^{2}}\left(1-{\left(\frac{r}{r_{1}}\right)}^{2}\right)$$

where $Q_{t}$ is the liquid solvent feed rate, *n* is the number of fibers, and *r*_1_ is the inner radius of the tubes.

The boundary conditions:

at z=0, $C_{CO_{2,t}}=0$ (the CO_2_ concentration in the fresh solvent is zero);

at z=L, $\frac{\partial C_{CO_{2},t}}{\partial z}=0$ (the convective flux is zero at the exit of the tube);

at r=0, $\frac{\partial C_{CO_{2,t}}\;}{\partial r}=0$ (axial symmetry assumption);

at $r=r_{1}$, $C_{CO_{2},t}=m\;C_{CO_{2},m}$ (at the gas-liquid interface, the solubility of CO_2_ in PG).

The forward reaction rate is expressed as follows [28].

(3) $$r_{CO_{2}}=-2.42\times 10^{16}\exp\left(\frac{-8544}{T}\right)\exp\left(0.44C_{Pg}\right)C_{Pg}C_{CO_{2}}$$

where *T*(K) is the liquid temperature, $C_{pg}$, are the concentrations of PG, and $C_{CO_{2}}$ is the concentration of CO_2_.

### 2.2. Membrane Skin

In this section, the model equations describe the unsteady steady-state gas diffusion across the membrane walls where the convective flux is neglected, and only diffusion is considered:

(4) $$\frac{\partial C_{i,m}}{\partial t}=-D_{i,m}\left[\frac{\partial }{\partial r}\left(\frac{1}{r}\frac{\partial C_{i,m}}{\partial r}\right)+\frac{\partial^{2}C_{i,m}}{\partial z^{2}}\right]$$

The boundary conditions of the membrane skin:

at z=0, $\frac{\partial C_{i},m}{\partial z}=0$ (at the inlet of the module, the membrane walls are solid, and diffusion flux is assumed insignificant);

at z=L, $\frac{\partial C_{i},m}{\partial z}=0$ (at the exit of the membrane module, the membrane walls are solid, and diffusion flux is ignored);

at $r=r_{1}$, $D_{i,m}\frac{\partial C_{i},m}{\partial r}=D_{i,t}\frac{\partial C_{i},t}{\partial r}$ (at the liquid-liquid interface, the fluxes are assumed equal);

at $r=r_{2}$, $C_{i,m}=C_{i,s}$ (at the membrane-gas interface, the membrane resistance is neglected, and the concentration of the gas in the shell side is assumed to be equal to that in the membrane walls).

### 2.3. The Shell of the Module (Gas Stream)

The unsteady steady-state gas concentration in the shell side:

(5) $$\frac{\partial C_{i,s}}{\partial t}=-D_{i,s}\left[\frac{\partial }{\partial r}\left(\frac{1}{r}\frac{\partial C_{i,s}}{\partial r}\right)+\frac{\partial^{2}C_{i,s}}{\partial z^{2}}\right]+v_{z,s}\left(\frac{\partial C_{i,s}}{\partial z}\right)$$

Here, $C_{i,s}$ is the molar concentration of the CO_2_ in the gas on the module shell side and $D_{is}$ is the diffusion coefficient of CO_2_ in the gas phase flowing on the shell side.

The perturbation of the gas velocity on the shell side [29] is modified to consider the sine wave frequency of the gas velocity on the shell side ($v_{sm}$), whereas $v_{sm}$ is the main gas velocity on the shell side before reaching the oscillating device.

(6) $$v_{z,s}=v_{zm}\;\sin\left(\omega t\right)\left\{1-{\left(\frac{r_{2}}{r_{3}}\right)}^{2}\right\}\left\{\frac{{\left(\frac{r}{r_{3}}\right)}^{2}-{\left(\frac{r_{2}}{r_{3}}\right)}^{2}-2ln\left(\frac{r}{r_{2}}\right)\;}{3+{\left(\frac{r_{2}}{r_{3}}\right)}^{4}-4{\left(\frac{r_{2}}{r_{3}}\right)}^{2}+4\ln\left(\frac{r_{2}}{r_{3}}\right)}\right\}$$

where ω is the frequency. The appropriate boundary conditions:

z=L, $C_{i,s}=C_{i,0}$ (the gas flows in the module counter currently; accordingly, the concentration of CO_2_ in the gas equals the initial concentration);

z=0, $\frac{\partial^{2}C_{i},sa}{\partial z^{2}}=0$ (the gas leaves from the entrances of the module and hence, convective flux of the exit gas stream is negligible);

$r=r_{2}$, $D_{i,s}\;\frac{\partial C_{i},sa}{\partial r}=D_{i,ms}\;\frac{\partial C_{i},ma}{\partial r}$ (at the membrane skin-shell gas interface, the diffusive flux is equal);

$r=r_{3}$, $\frac{\partial C_{i},sa}{\partial r}=0$ (at the outer module radius, the diffusion flux is neglected).

The radius of the free surface ($r_{3}$), is expressed as follows:

(7) $$r_{3}=r_{2}{\left(\frac{1}{1-\varphi }\right)}^{0.5}$$

The module void fraction (φ):

(8) $$\varphi =\frac{R^{2}-n\;r_{2}^{2}}{R^{2}}$$

R, and $r_{2}n$ are the inner radius of the module and fiber outer radius, respectively. *Q_t_* is the solvent circulation volumetric rate, and n is the number of fibers. Table 1 lists the characteristics of the hollow fiber membrane module. The experimental conditions were at atmospheric pressure and room temperature.

## 3. Mass Transfer Coefficient, $K_{G}$

The total mass transfer coefficient, $K_{G}\left(\frac{kmol}{m^{2}.s.kPa}\right)$, was estimated based on the two-phase theory as the ratio of the CO_2_ absorption flux ($J_{CO_{2}}$) to the solute concentration gradient between gas and liquid [30]. CO_2_ absorption flux ($J_{CO_{2}}$) was calculated based on the following expression [31]:

(9) $$J_{CO_{2}}\left(mol/m^{2}.s\right)=\frac{Q_{g,in}\;y_{CO_{2},in}-Q_{g,out}\;y_{CO_{2}}{,}_{out}}{A}$$

where $Q_{g}$ is the gas molar flow rate (mol/s) (assuming inlet and exit flow rate are the same; neglecting the effect of the amount of CO_2_ being absorbed on the exit gas flow rate), $y_{CO_{2},in}$ and $y_{CO_{2},out}$ are the inlet and exit mole fractions of CO_2_, respectively, and *A* is the gas-–liquid interface contact area based on the internal diameter of the hollow fiber. The total mass transfer coefficient is calculated as follows [32]:

(10) $$K_{G\;}\left(m/s\right)=\frac{J_{CO_{2}}}{C_{g,Lm}}$$

where $K_{G}$ symbolizes the overall mass transfer coefficient in the gas phase and $C_{g,Lm}$ is the average log mean concentration of CO_2_ in the bulk gas phase of the outlet and inlet concentration [33].

(11) $$C_{g,lm}=\frac{C_{CO_{2},in}-C_{CO_{2},out}}{\ln\left(C_{CO_{2},in}/C_{CO_{2},out}\right)}$$

The exit gas concentration ($C_{CO_{2},out}$) is determined using the boundary line integration built using the software Comsol, version 6.0 [34].

## 4. Results and Discussion

### 4.1. Model Validation

**Figure 2:**
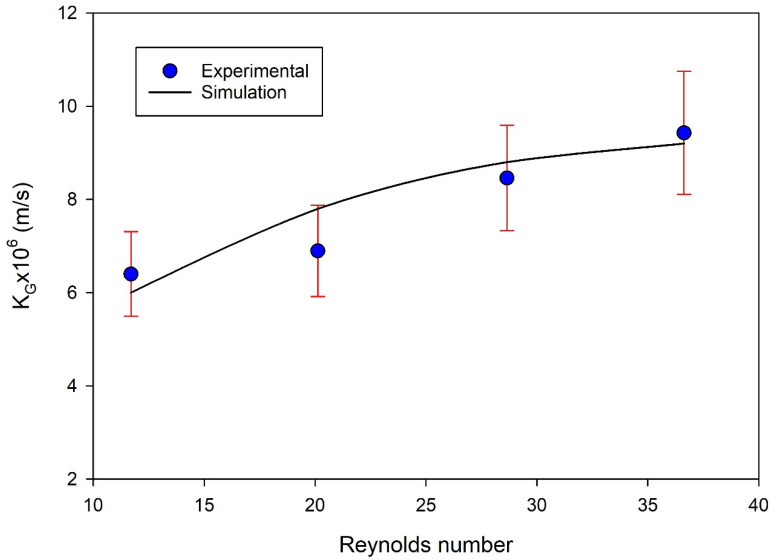
Model validation with the experimental data [35]. The gas phase is fed into the lumen side and the solvent into the shell side (Figure 1a).

The model predictions were validated using experimental data available from the literature [35]. The model validation was performed by comparing the overall mass transfer coefficient between the experimental data and simulation predictions. As per the experimental data used in the model validation, the gas stream was fed to the lumen side and the solvent to the shell side (Figure 1a). Figure 2 shows the effect of the feed flow rate represented by the Reynolds number on the overall mass transfer coefficient (*K_G_*). The results revealed that the mass transfer coefficient increased with an increased Reynolds number, a common trend of the membrane contactors that is attributed to the increase in gas velocity being associated with a decrease in the boundary layer thickness. This replicates the presence of supplementary mixing in the gas phase boundary layer, as the oscillating flow disrupts the thickness of the boundary layer and the flow regime around this layer [36]. The model predictions revealed that increasing the gas velocity had significant effects on the total gas mass transfer coefficients owing to the formation of a thin film for mass transfer layer at higher speeds, which resulted in increases in the mass transfer coefficient of the gas phase layer in spite of decreasing the residence time of the gas inside the membrane module at high velocities. Increase in the velocity has insignificant impact on the overall mass transfer coefficient [37].

**Figure 3:**
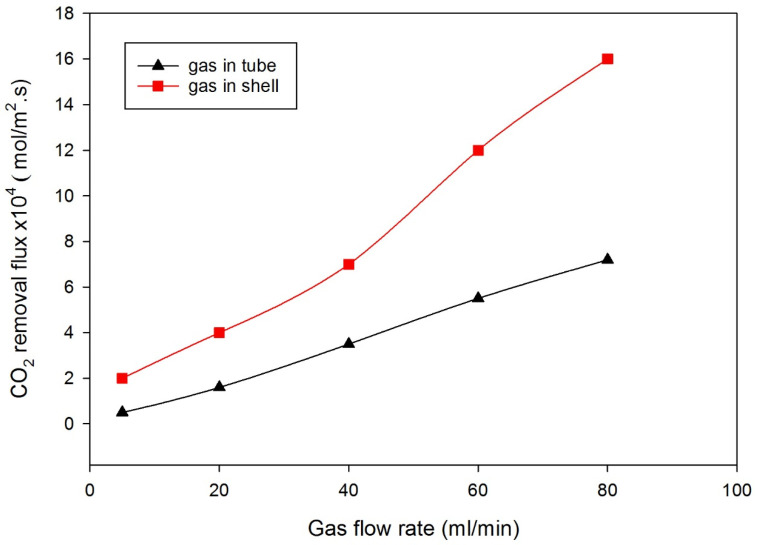
Comparison between the oscillatory gas flows in the shell and tube sides at operating conditions (temperature of 25 °C, atmosphere pressure, liquid flow rate of 10 mL/min).

A comparison of the gas feed rate between the tube side and shell side revealed that introducing the gas to the shell side of the membrane module enhanced the overall mass transfer coefficient compared with gas being introduced into the tube side, attributed to increases in surface contact area and gas residence time in the membrane module (Figure 3). Accordingly, the rest of the results are based on gas flow fed into the shell side and liquid solvent into the tube side (Figure 1b). This configuration matches most of the experimental work performed in the literature, where liquids are introduced to the lumen side of the membrane module for better contact area and residence time [8,33]. The increase in the mass transfer coefficient when gas is fed into the shell side is attributed to better mixing and the increase in residence time when the gas flow rate decreases. Our simulation is based on the data available in Table 1, where the gas is introduced into the shell side.

### 4.2. Gas Feed Perturbation

**Figure 4:**
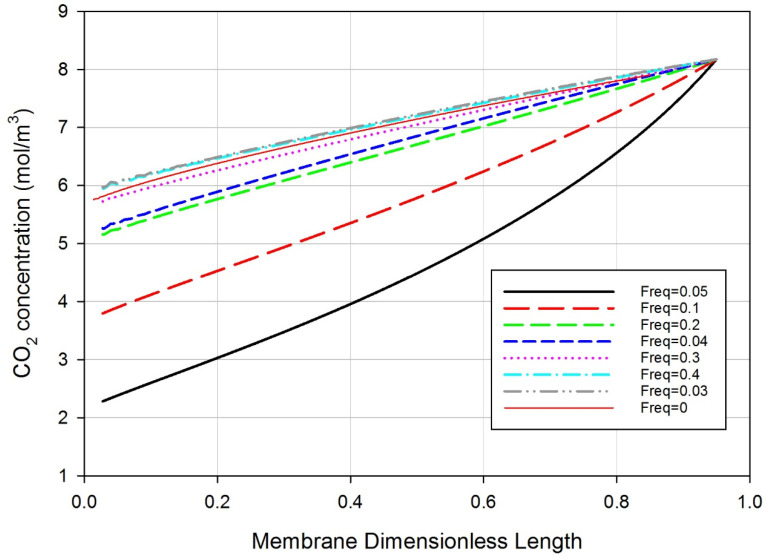
Effect of gas feed oscillation frequency on the CO_2_ concentration profile versus membrane dimensionless length of the gas flow on the shell side. Gas feed rate 500 mL/min, liquid feed rate 10 mL/min.

The gas feed velocity coefficient at different employed sinusoidal frequencies was investigated. As absorption occurred in a gas-liquid membrane system that was stabilized very rapidly, a time range of 60 s was investigated [35]. The frequency range was maintained while increasing the speed gradually and reaching the maximum point in 60 s. For example, at a frequency of 0.03, the gas speed gradually increased until it reached its maximum value. Frequencies (other than 0.05) cause the velocity to reach the maximum speed more than once in an oscillatory manner within the studied range. Under sinusoidal operating conditions, the velocity of the feed gas at different frequencies is described in Figure 4. The figure shows the effect of the feed gas sinusoidal frequency on CO_2_ concentration along the shell side of the membrane unit. The lowest exit CO_2_ concentration profile was at a frequency of 0.05 HZ. A smaller CO_2_ concentration in the exit stream indicates a higher percentage removal of CO_2_ from the gas stream mixture [35].

**Figure 5:**
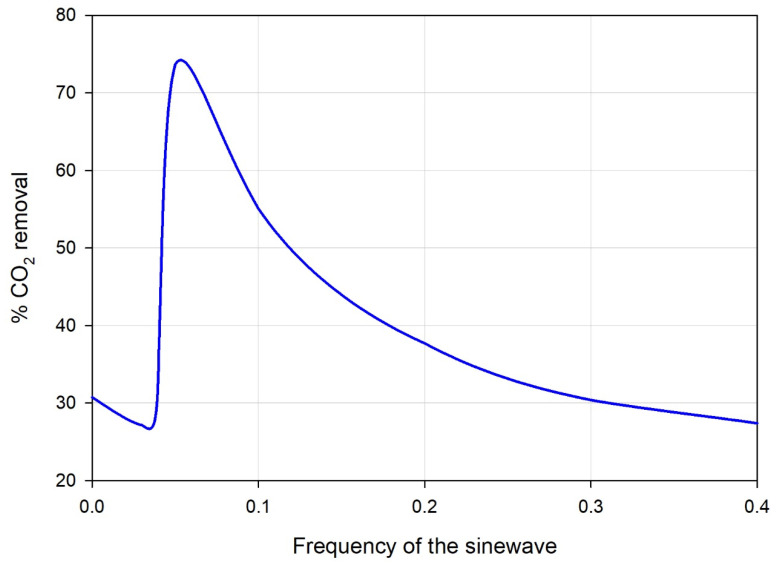
Effect of the sinewave frequency in the feed stream on the percent removal of carbon dioxide from the natural gas stream. The liquid feed rate is 10 mL/min; the gas feed rate is 500 mL/min.

Figure 5 depicts the effect of sinewave frequencies on the percent removal of CO_2_ from the feed gas stream. The figure shows that the CO_2_ removal percentage was low (0.03 percent), and the removal rate reached the maximum at a frequency of 0.05. Further increase in the frequencies causes a decline in the percentage of CO_2_ removal, and that is attributed to the sinusoidal number of cycles; at a frequency of 0.2, the velocity coefficient increases within the investigated interval of 60 s around four times. The results indicated that an oscillation frequency of 0.05 achieves the highest CO_2_ removal rate associated with a reduction in the boundary layer thickness. This reflected the presence of supplementary mixing in the gas phase boundary layer as the oscillating flow interrupted the boundary layer thickness and flow regime around this layer [37].

The increase in membrane efficiency caused by a frequency of 0.05 shows the best removal rate as the velocity reaches a maximum and then decreases to give more room for fresher gas and increase the driving force. Further increase in frequencies leads to a greater number of cycles within the investigated period, decreasing the residence time and not giving enough time for separation [35]. The reasonable frequency of 0.05 doubled the percentage removal because the pressure wave generated by the oscillation of the feed gas produced localized increases in the partial pressure driving force for mass transfer across the membrane. The results were consistent with previous experimental observations [37].

**Figure 6:**
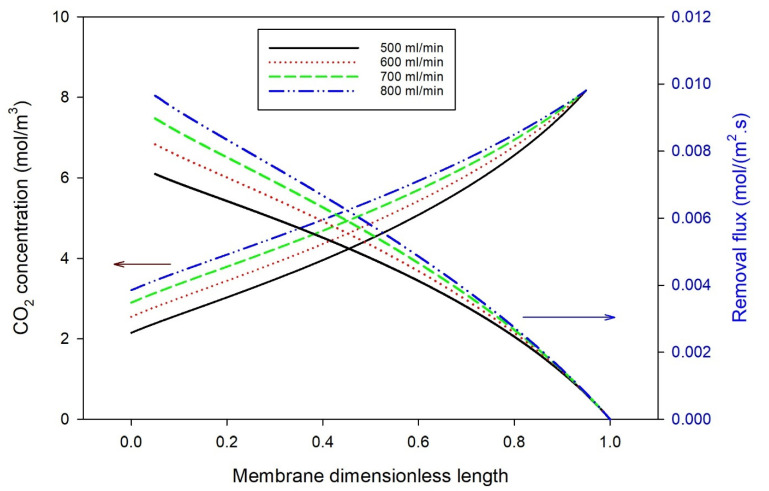
Effect of feed gas flow rate on CO_2_ concentrations and removal flux at the optimum frequency of 0.05.

Figure 6 shows the model predictions for the effect of a feed gas flow rate at a constant sinusoidal frequency of 0.05 on CO_2_ concentrations and removal flux along the dimensionless membrane length. The figure shows that, along the membrane dimensionless length, the higher the gas flow rate, the lower the CO_2_ removal rate, and the higher the CO_2_ removal flux. This is attributed to the fact that an increase in the gas flow rate usually reduces the thickness of the gas boundary layer, which is supposed to enhance the mass transfer rate and increase the CO_2_ removal ratio. At the same time, an increase in the feed gas flow rate reduces the residence time, which reduces the rate of mass transfer, and thus, reduces the rate of CO_2_ absorption along with the membrane gas-phase compartment (membrane unit envelope side). Similar results were reported in previous studies [38,39,40]. This indicates that the residence time strongly impacts the CO_2_ removal rate. The increase in the CO_2_ removal flux with the gas flow rate is credited to the increased amount of CO_2_ being absorbed with the gas flow rate. Accordingly, residence time strongly affects CO_2_ removal compared with gas mass transfer intensification.

**Figure 7:**
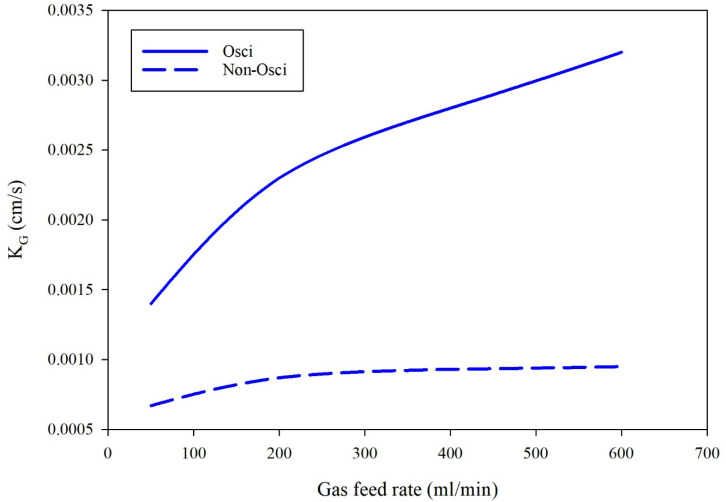
Effect of gas feed rate on the CO_2_ overall mass transfer coefficients under the optimum oscillating frequency (0.05) and in normal conditions at a constant liquid feed rate of 10 mL/min. Gas was introduced to the shell side and solvent into the tube side.

Figure 7 indicates the influence of the gas feed rate on the total mass transfer coefficients under the presence (0.05 sinusoidal frequency) and absence of feed oscillation. The total mass transfer coefficient increased with the gas feed rate due to the amount of carbon dioxide absorbed, and the thickness of the gas boundary layer decreased with the increase in gas velocity. The total mass transfer coefficients increase with the increase in gas velocity mentioned earlier. The results are within the range of those obtained experimentally. In contrast, the effect of gas supply rate on the total mass transfer coefficient in the absence of gas supply disturbance in the sine waveform is negligible. There is a significant increase in K_G_ compared with the non-oscillating mode of operation. The oscillating flow disturbed the boundary layer thickness and flow regime around the layer, causing further mixing in the gas phase boundary layer, and hence, enhancing the CO_2_ diffusion rate to the absorbing solvent.

**Figure 8:**
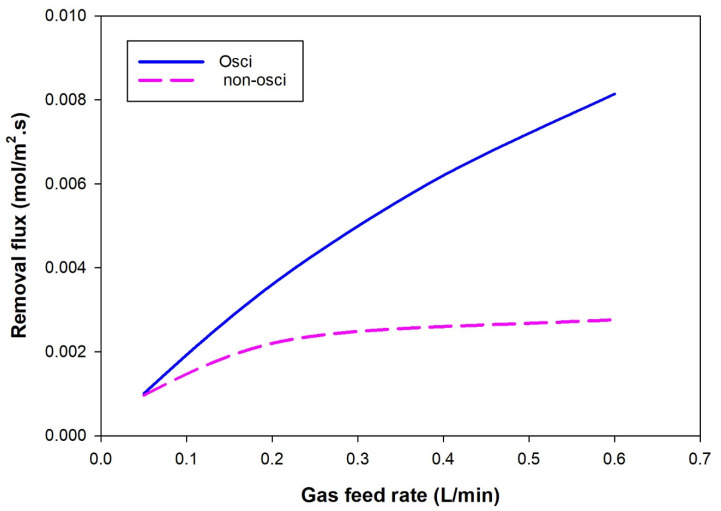
Influence of feed perturbation on the CO_2_ removal flux under the effect of oscillatory and non-oscillatory gas feed rate. The oscillating mode was at the optimum frequency of 0.05 HZ.

Figure 8 compares the CO_2_ removal flux between the oscillatory and non-oscillatory gas feed velocity represented by the gas feed rate. The figure shows that the removal percentage increased slightly in the non-oscillating operating mode at a gas feed rate up to a flow rate of 0.25 L/min. Beyond this, the change in the removal ratio with the gas feed rate was insignificant. In contrast, under oscillating mode, a considerable increase in the mass transfer coefficient was observed, indicating the success of the feed gas oscillation mode of operation.

## 5. Conclusions

A two-dimensional (2D) transient mathematical model, assumed to operate under non-wetted mode conditions, was upgraded by considering feed gas oscillation conditions. The feed gas enters the membrane module at different sinusoidal waves with different frequencies. The model was utilized to study the absorption of CO_2_ from natural gas into a PG aqueous solution within a hollow fiber membrane unit. In addition to the perturbation of the inlet gas velocity, the model considers the radial and axial diffusion within the hollow fiber lumen, membrane walls, and module shell sides. The predicted results revealed that the feed gas oscillation frequency and flow rate of both gas and liquid streams influenced CO_2_ removal efficiency. We found significant improvement in the sinusoidal frequency of the feed gas stream. The optimum frequency that doubled the percentage removal of CO_2_ was around 0.05. The percentage removal increased from almost 30% at no oscillation to around 70% at a frequency of 0.05. The model predictions were in good agreement with experimental data.

## Figures and Tables

**Table 1:** Membrane characteristics used in the mathematical model at room temperature and ambient pressure.

Number of fibers	20
Hollow fiber, inner radius, mm	0.21
Hollow fiber, outer radius, mm	0.55
Module inside radius, m	0.008
Module length, m	0.25

## Data Availability

All data generated or analyzed during this study are included in this published article. Additional explanations or data are available from the corresponding author upon reasonable request.
